# Normalized Cardiac Structure and Function in COVID-19 Survivors Late After Recovery

**DOI:** 10.3389/fcvm.2021.756790

**Published:** 2021-11-29

**Authors:** Yi-Ping Gao, Wei Zhou, Pei-Na Huang, Hong-Yun Liu, Xiao-Jun Bi, Ying Zhu, Jie Sun, Qiao-Ying Tang, Li Li, Jun Zhang, Rui-Ying Sun, Xue-Qing Cheng, Ya-Ni Liu, You-Bin Deng

**Affiliations:** Department of Medical Ultrasound, Tongji Hospital, Tongji Medical College, Huazhong University of Science and Technology, Wuhan, China

**Keywords:** COVID-19, speckle tracking echocardiography, myocardial strain, NT-proBNP, troponin

## Abstract

**Background:** Coronavirus disease 2019 can result in myocardial injury in the acute phase. However, information on the late cardiac consequences of coronavirus disease 2019 (COVID-19) is limited.

**Methods:** We conducted a prospective observational cohort study to investigate the late cardiac consequences of COVID-19. Standard echocardiography and myocardial strain assessment were performed, and cardiac blood biomarkers were tested in 86 COVID-19 survivors 327 days (IQR 318–337 days) after recovery. Comparisons were made with 28 age-matched and sex-matched healthy controls and 30 risk factor-matched patients.

**Results:** There were no significant differences in all echocardiographic structural and functional parameters, including left ventricular (LV) global longitudinal strain, right ventricular (RV) longitudinal strain, LV end-diastolic volume, RV dimension, and the ratio of peak early velocity in mitral inflow to peak early diastolic velocity in the septal mitral annulus (E/e') among COVID-19 survivors, healthy controls and risk factor-matched controls. Even 26 patients with myocardial injury at admission did not have any echocardiographic structural and functional abnormalities. There were no significant differences among the three groups with respect to serum concentrations of N-terminal pro-B-type natriuretic peptide (NT-proBNP) and high-sensitivity cardiac troponin I (cTnI).

**Conclusion:** This study showed that COVID-19 survivors, including those with myocardial injury at admission and those with severe and critical types of illness, do not have any echocardiographic evidence of cardiac structural and functional abnormalities 327 days after diagnosis.

## Introduction

Coronavirus disease-2019 (COVID-19) is now the deadliest pandemics caused by the novel severe acute respiratory syndrome-coronavirus-2 (SARS-CoV-2) (1). Though it primarily affects the respiratory system, cardiovascular complications are common in COVID-19 (2, 3). Myocardial injury reflected through elevated troponin concentration was reported in the acute stage of COVID-19 (4, 5). Left ventricular (LV) and right ventricular (RV) enlargements and dysfunctions were found with conventional and speckle tracking echocardiography in patients with COVID-19 (6–8). Since most COVID-19 patients recover from the illness, the understanding of the late cardiovascular consequences of infection was important. Until now, there are only a few studies on the cardiac outcome of COVID-19 survivors (9–13). These studies have reported residual cardiac structural and functional abnormalities even after recovery from COVID-19 using cardiac magnetic resonance (CMR) imaging (11–13) and echocardiography (9–11). However, these studies have been limited by their short time interval between COVID-19 diagnosis and follow-up study from 26 to 140 days which may not be long enough for cardiac abnormalities to resolve. Therefore, we performed the present study to examine the myocardial mechanical function with speckle tracking echocardiography as well as cardiac blood biomarkers in COVID-19 survivors 327 days after diagnosis.

## Methods

### Study Design and Participants

This is a single-center, prospective observational cohort study undertaken in Tongji Hospital of Huazhong University of Science and Technology, a designated medical unit for treating patients with COVID-19. COVID-19 survivors were identified from the hospital medical record system and recruited through posting recruitment notices. Exclusion criteria were unwillingness to participate, incapability of communication, acute conditions such as infection, organ dysfunction and active autoimmune disease, and other illness requiring hospitalization. Patients with unsatisfactory recordings of echocardiograms were also excluded. Finally, a total of 86 consecutive patients with a history of confirmed SARS-CoV-2 infection using reverse transcription-polymerase chain reaction swab test of the upper respiratory tract were recruited between December 2020 and January 2021. In total, 28 healthy subjects matched for age and sex were recruited as the healthy controls. While the other 30 matched for age, sex, hypertension, diabetes mellitus, smoking, hypercholesterolemia, and coronary artery disease were also recruited as the risk factor-matched controls. All control group subjects were recruited from communities with consent of each participant. Our research was in concordance with the Declaration of Helsinki and the International Conference on Harmonization of Good Clinical Practice. The Tongji Hospital Ethics Committee approved the study (TJ-C20200156) and informed consent was obtained from each participant before their enrollment in the study.

Clinical characteristics, laboratory test results, and treatment for the acute phase of illness were collected from electronic medical records or patient discharge summaries. After recording the present clinical characteristics, all subjects underwent blood sampling, standard echocardiography, and myocardial strain assessment.

### Standard Echocardiography and Myocardial Strain Assessment

All participants underwent echocardiographic examinations according to the recommendation of the American Society of Echocardiography using a Vivid E95 digital ultrasound system (GE Medical System, Horten, Norway) equipped with a 1.7–3.4 MHz M5Sc phased array transducer (14). All images were analyzed offline using commercially available software (EchoPac version 203, GE Vingmed, Horten, Norway). LV dimension, wall thickness, and LV mass were obtained from M-mode echocardiography. The biplane Simpson's method was used to calculate LV volume and ejection fraction. Left atrial (LA) volume was measured with the modified Simpson's method. LA volume and LV volume, and mass were indexed to the body surface area. Peak early (E) and late diastolic (A) velocities in mitral inflow, and peak early diastolic velocity (e') in septal mitral annulus were measured, and the E/A and E/e' ratios were calculated. Each parameter was averaged in three cardiac cycles.

Right atrial and RV dimensions and RV area were measured in the apical four-chamber view. RV fractional area change was calculated by dividing the difference between RV end-diastolic and end-systolic areas by the end-diastolic area. The tricuspid annular plane systolic excursion was obtained from the M-mode recording as the systolic displacement of the tricuspid lateral annulus. Tricuspid lateral annular systolic tissue velocity was measured in apical four-chamber view. The presence and severity of tricuspid regurgitation and pulmonary artery systolic pressure were assessed on color Doppler and continuous wave Doppler spectrum according to current guidelines.

Myocardial strain off-line analysis was performed using software (EchoPac version 203, GE Vingmed, Horten, Norway) on the two-dimensional gray-scale image with a frame rate of 70–90 frames/s according to the recommendations of the American Society of Echocardiography and the European Association of Cardiovascular Imaging (15). LV myocardial strain was obtained from the apical four-chamber, apical two-chamber, apical long-axis using a 17-segmental model with speckle tracking echocardiographic method. The LV global longitudinal strain was calculated by averaging peak strain values in 17 LV segments. RV free wall longitudinal strain for basal, mid, and apical segments was obtained in the apical four-chamber view. RV longitudinal strain was calculated by averaging the peak strain values in the three segments of the RV free wall.

### Laboratory Examination

Peripheral venous blood samples were drawn at least 30 min before echocardiographic examination. Blood samples were processed using standardized commercially available test kits for analysis of high-sensitivity troponin I [(cTnI), Roche Diagnostics, Rotkreuz, Switzerland] and N-terminal pro-B-type natriuretic peptide [(NT-proBNP), Abbott, Illinois, USA]. Myocardial injury was defined as a serum cTnI above the upper 99th percentile value. Serum NT-proBNP level was considered elevated according to the age-specific diagnostic threshold for heart failure. The local laboratory cTnI values above the upper 99th percentile counted as a significant increase were 15.6 pg/ml for women and 34.2 pg/ml for men. The age-specific diagnostic thresholds of serum NT-proBNP for heart failure were as follows: <62.9 pg/ml for men and <116 pg/ml for women (18–44 years old); <89.3 pg/ml for men and <169 pg/ml for women (45–54 years old); <161 pg/ml for men and <247 pg/ml for women (55–64 years old); <241 pg/ml for men and <285 pg/ml for women (65–74 years old); <486 pg/ml for men and <738 pg/ml for women (above 75 years old).

### Statistical Analysis

Statistical analysis was carried out using SPSS version 21 software (IBM, Armonk, NY, USA). Normality was evaluated using the Shapiro-Wilk test. Categorical variables were expressed as counts and percentage, and continuous variables as mean ±*SD* or median [interquartile range (IQR)]. Wilcoxon test was utilized for comparisons of the data obtained at the acute phase and recovery of the illness. Unpaired Student's *t*-test was used to compare clinical data between two groups if normally distributed, and Mann-Whitney *U*-test if not normally distributed. Comparisons among three groups were performed using one-way ANOVA with Bonferroni corrected *post-hoc* comparisons for normal distribution or Kruskal-Wallis tests for non-normal distribution, as appropriate. Differences in proportions were analyzed with the Chi-square test or the Fisher exact test. A *p*-value < 0.05 was considered to indicate statistical significance.

## Results

### Patient Characteristics

A total of 86 patients were enrolled in this study (Table 1). Median (IQR) age was 58 (39–70) years and 32 (37%) were men. Among the 86 patients, 45 (52%) were diagnosed as having moderate-type COVID-19 illness, 27 (31%) as having severe-type, and 14 (17%) as having critical-type from January to February 2020 according to the Diagnosis and Treatment Protocol of Novel Coronavirus issued by the National Health Commission of the People's Republic of China.^1^ Furthermore, 78 (91%) patients required hospitalization. Among these 78 hospitalized patients, 1 patient (1%) underwent extracorporeal membrane oxygenation, 6 (8%) underwent mechanical ventilation, and 10 (13%) underwent non-invasive ventilation with positive airway pressure. Nasal cannula oxygen support was needed in 68 (87%) patients. All patients received antiviral and antibiotics therapy. Corticosteroid was used in 41 of 78 hospitalized patients (53%). Histories of cardiovascular conditions included hypertension in 32 (37%) patients, diabetes mellitus in 14 (16%), hypercholesterolemia in 16 (19%), and coronary heart disease in 13 (15%). During hospitalization, serum cTnI and NT-proBNP levels were available in 64 and 45 patients, respectively. Among them, a significant rise in cTnI was detected in 26 patients (26/64, 41%) while an elevated NT-proBNP level was found in 25 patients (25/45, 56%)

**Table 1:** Clinical characteristics, echocardiographic findings, and laboratory results of coronavirus disease 2019 (COVID-19) survivors 327 days after diagnosis.

	**Healthy control**	**Risk factor-matched control**	**COVID-19**	***p*-value**
	**(*n* = 28)**	**(*n* = 30)**	**(*n* = 86)**	
**Patient characteristics**				
Age, years	56 (37–65)	62 (39–67)	58 (39–70)	0.392
Male, *n*%	10 (36%)	11 (37%)	32 (37%)	0.990
Body mass index, kg/m^2^	23 ± 3	24 ± 3	24 ±3	0.304
Body surface area, m^2^	1.7 ± 0.2	1.7 ± 0.2	1.7 ± 0.2	0.561
Heart rate, beats/min	67 (61–81)	69 (63–73)	73 (65–79)	0.119
Systolic blood pressure, mm Hg	125 ± 12	126 ± 16	131 ± 18	0.132
Diastolic blood pressure, mm Hg	73 (67–82)	72 (67–79)	77 (70–82)	0.228
Oxygen saturation, %	NA	NA	98 (97–99)	NA
Hypertension, *n*%	0 (0%)	10 (33%)^*^	32 (37%)^*^	0.001
Diabetes mellitus, *n*%	0 (0%)	2 (7%)	14 (16%)^*^	0.032
Coronary heart disease, *n*%	0 (0%)	3 (10%)	13 (15%)	0.076
Hypercholesterolemia, *n*%	0 (0%)	9 (30%)^*^	16 (19%)^*^	0.003
**Echocardiographic findings**				
LA dimension, mm	31 (28–33)	31 (28–33)	32 (29–34)	0.388
LV dimension, mm	45 (43–50)	45 (43–49)	46 (44–49)	0.780
IVS thickness, mm	8 (7–8)	8 (7–9)	8 (7–9)	0.180
LV posterior wall thickness, mm	8 (7–8)	8 (7–8)	8 (7–9)	0.094
LV mass, g/m^2^	73 (63–87)	78 (64–86)	80 (67–96)	0.346
LV end-diastolic volume, ml/m^2^	47 (43–51)	48 (44–52)	45 (40–54)	0.866
LV end-systolic volume, ml/m^2^	18 (15–19)	17 (15–19)	17 (14–21)	0.889
LV ejection fraction, %	63 (61–67)	63 (61–67)	63 (61–68)	0.870
LA volume, ml/m^2^	22 (18–26)	22 (18–27)	21 (18–25)	0.750
E/A ratio	1.1 (0.8–1.4)	1.1 (0.8–1.2)	0.9 (0.7–1.3)	0.190
E/e' ratio	8 ± 3	9 ± 4	9 ± 2	0.426
LV GLS, %	21 ± 2	21 ± 2	20 ± 2	0.381
LV GLS < 16%, *n*%	0 (0%)	0 (0%)	4 (5%)	0.476
RA dimension, mm	34 (30–36)	34 (30–35)	33 (30–38)	0.554
RV dimension, mm	31 (27–33)	30 (27–34)	32 (28–36)	0.217
TAPSE, mm	27 (24–29)	26 (23–28)	26 (24–28)	0.346
RV fractional area change, %	47 ± 9	49 ± 8	51 ± 9	0.158
S', cm/s	14 (13–17)	14 (13–17)	14 (13–16)	0.936
PASP, mm Hg	23 (19–28)	24 (19–28)	25 (21–30)	0.707
RV longitudinal strain, %	30 ± 5	30 ± 6	29 ± 6	0.722
RV longitudinal strain < 20%, *n*%	1 (4%)	1 (3%)	2 (2%)	1.000
Pericardial effusion, *n*%	0 (0%)	0 (0%)	1 (1%)	1.000
**Laboratory results**				
NT-proBNP, pg/mL	36 (15–65)	41 (19–72)	51 (24-104)	0.113
cTnI, pg/mL	1.9 (1.9–2.5)	1.9 (1.9–2.8)	1.9 (1.9–4.9)	0.159

*Numbers are given as median (interquartile range) or mean ± standard deviation or as case numbers with percentages in parentheses*.

*NA, not applicable; LA, left atrium; LV, left ventricle; IVS, interventricular septum; E, peak early diastolic velocity in mitral inflow; A, late diastolic velocity in mitral inflow; e', peak early diastolic velocity in septal mitral annulus; GLS, global longitudinal strain; RA, right atrium; RV, right ventricle; TAPSE, tricuspid annular plane systolic excursion; S', tricuspid lateral annular systolic tissue velocity; PASP, pulmonary artery systolic pressure; NT-proBNP, N-terminal pro-B-type natriuretic peptide; cTnI, high-sensitivity cardiac troponin I*.

* *p < 0.01, vs. healthy control*.

Patient characteristics, echocardiographic findings, and cardiac biomarkers on the day of echocardiographic strain are shown in Table 1. The median (IQR) interval between the COVID-19 diagnosis and echocardiographic examination was 327 (318–337) days. Exertional shortness of breath and chest discomfort was reported in 25 (29%) and 33 (38%), respectively, on the day of echocardiographic examination.

### Echocardiographic Findings

No difference was found among COVID-19 survivors, healthy controls, and risk factor-matched patients with respect to age, percentage of male subjects, body mass index, body surface area, heart rate, and blood pressure. Hypertension, diabetes mellitus, coronary artery disease, and hypercholesterolemia were more common in COVID-19 survivors than those in healthy controls, but there were no differences between COVID-19 survivors and risk factor-matched patients (Table 1).

**Figure 1:**
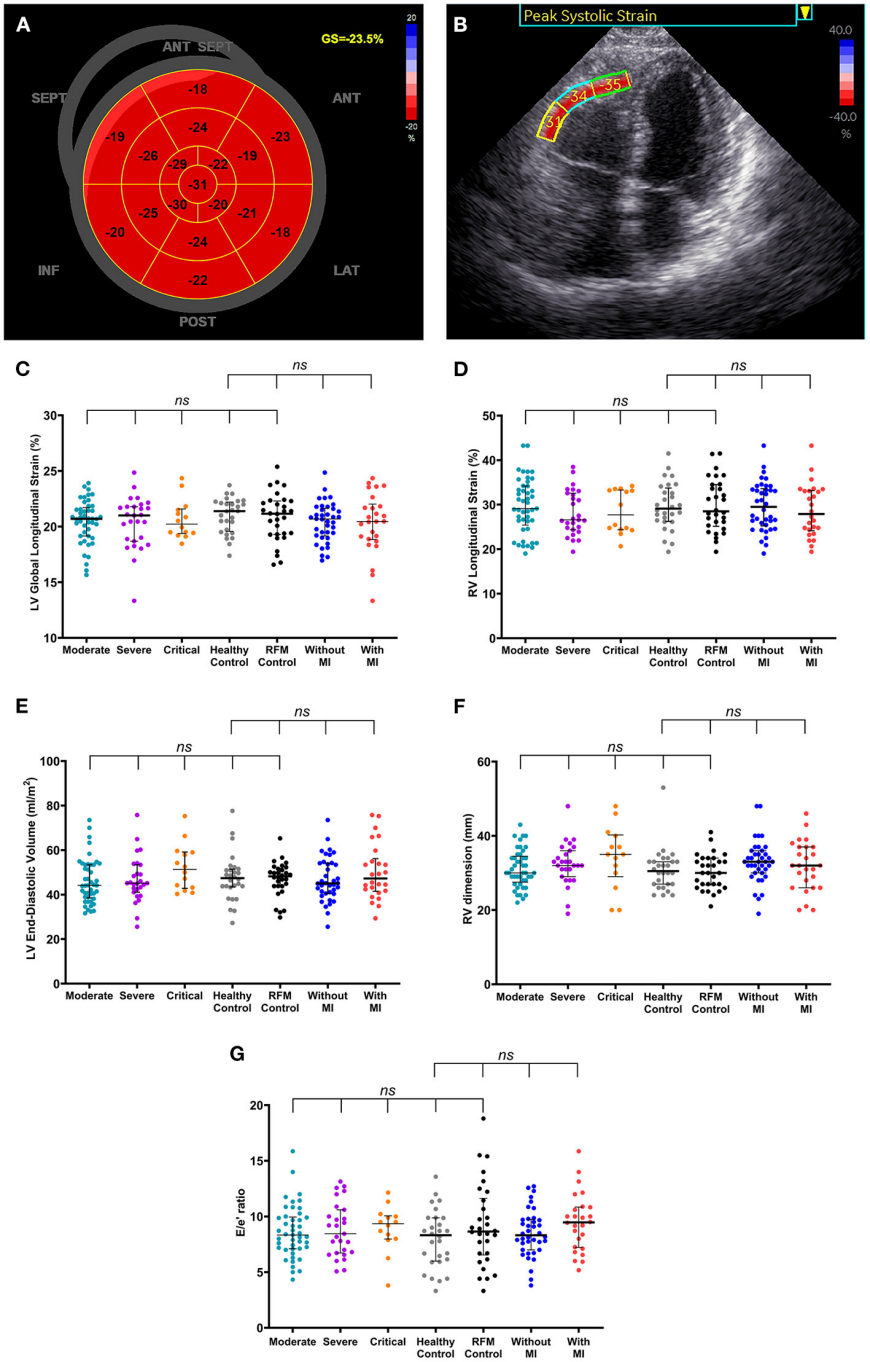
Normalized cardiac structure and function in coronavirus disease 2019 (COVID-19) survivors late after the recovery. **(A,B)** A patient (75–80 years old) with no history of hypertension, diabetes, and/or coronary heart disease was diagnosed with severe-type COVID-19 illness. High-sensitivity troponin I level was 1,137 pg/ml at admission and 4.3 pg/ml on the day of echocardiographic examination (316 days after COVID-19 diagnosis). **(A)** Shows normal left ventricular (LV) global longitudinal strain (GS) and panel B shows normal right ventricular (RV) free wall longitudinal strain for basal, mid, and apical segments. **(C–G)** There were no significant differences in LV global longitudinal strain **(C)**, RV longitudinal strain **(D)**, LV end-diastolic volume **(E)**, RV dimension **(F)**, and the ratio of peak early velocity in mitral inflow to peak early diastolic velocity in the septal mitral annulus [E/e', **(G)**] among groups classified according to disease severity and the presence of myocardial injury at admission, healthy control, and risk-matched control. Longer black lines indicate the medians and shorter black lines indicate the interquartile ranges. Each dot represents a value. ANT, anterior; LAT, lateral; POST, posterior; INF, inferior; SEPT, septum; ANT SEPT, anterior septum; RFM, risk-factor matched; MI, myocardial injury; LV, left ventricular; RV, right ventricular.

There were no significant differences in all echocardiographic structural and functional parameters, including LV global longitudinal strain, RV longitudinal strain among COVID-19 survivors, healthy controls, and risk factor-matched controls (Figures 1A,B, Table 1). There were even no significant differences in echocardiographic structural and functional parameters among groups classified according to disease severity and the presence of myocardial injury at admission, healthy control, and risk-matched control (Figures 1C–G).

### Blood Biomarkers

**Figure 2:**
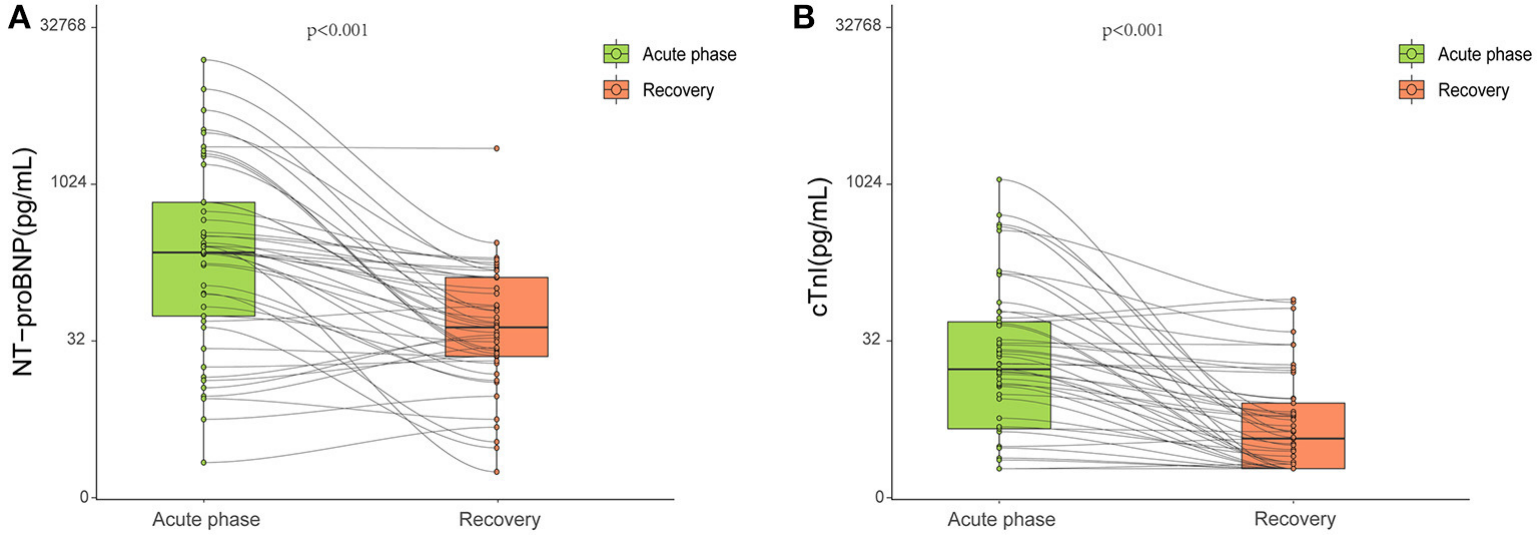
Blood biomarkers obtained at the acute phase and late after the recovery. During hospitalization, serum NT-proBNP **(A)** and cTnI **(B)** levels were available in 45 and 64 patients, respectively. Both were significantly decreased 327 days after diagnosis compared with those in the acute phase (*p* < 0.001). Each small circle represents a value. The top and bottom of the rectangle represent the interquartile range. Bold black lines in the rectangle indicate medians.

There were no significant differences among the three groups with respect to serum concentrations of NT-proBNP and cTnI (Table 1). In a proportion of survivors with obtainable data in the acute phase, NT-proBNP and cTnI concentrations were both significantly decreased 327 days after diagnosis when compared with those in the acute phase (Figure 2).

## Discussion

Our study showed that there were no significant differences in echocardiographic structural and functional parameters among COVID-19 survivors, healthy control, and risk factor-matched control 327 days after diagnosis regardless of the presence of myocardial injury in the acute phase and severity of the illness at admission. In addition, blood biomarkers of myocardial injury and function revealed no significant differences among COVID-19 survivors, healthy and risk-factor matched controls.

Coronavirus disease 2019 is a global pandemic leading to high morbidity and mortality (1). A significant proportion of patients with COVID-19 were reported to suffer from a myocardial injury in the acute phase. Echocardiographic abnormalities, including global LV dysfunction, regional wall motion abnormalities, diastolic dysfunction, RV dysfunction, and pericardial effusion were detected in patients with COVID-19 in the acute phase and a higher prevalence of echocardiographic abnormalities was found in patients with biomarker evidence of myocardial injury (4). CMR also revealed myocarditis, LV dysfunction, pericarditis, and Takotsubo cardiomyopathy in the acute phase of COVID-19 illness, indicated by abnormalities in T1 and T2 mapping and late gadolinium enhancement images (16–18). Nevertheless, it is still unclear whether the myocardial injury at the acute phase of illness leaves persistent lesions and how significant these abnormalities are in the long run. A few studies on the cardiovascular consequences of COVID-19 with limited follow-up intervals have been published (9–13, 19–24). In a study of cohort patients 71 days after recovery of COVID-19, magnetic resonance revealed cardiac involvement, including myocardial late gadolinium enhancement, raised myocardial native T1 and T2 in 78% of patients independent of preexisting conditions, severity, and overall course of the acute illness (12). Echocardiographic studies showed similar findings. The study of Zhou et al. reported LV dysfunction with decreased LV ejection fraction after a short period of 1–4 weeks following discharge (20). Another study showed that despite normalized blood concentrations of troponin and NT-proBNP, 29% of survivors had an abnormality in echocardiography after 3 months of admission, with reverse RV remodeling in the majority reflected by dilated RV dimension and decreased RV fractional area change (9). To notice, 80% of patients in this study had undergone mechanical ventilation, indicating severely impaired pulmonary structure and function. Thus, the above observed persistent RV dysfunction could not simply be attributed to direct myocardial injury. Preservation in cardiac consequence has been reported (10, 21–23). The study of Catena et al. reported no structural and functional sequelae in the heart of survivors of COVID-19 more than 1 month after recovery from illness (10). Daher et al. also demonstrated no echocardiographic impairments in 33 patients with severe illness after 6 weeks following discharge (23). However, these studies were limited by their short time periods at follow-up, leaving long-term cardiovascular consequences of COVID-19 poorly understood. In the present prospective study, COVID-19 survivors were evaluated after a relatively long time period with a median interval of 327 days after diagnosis, and no elevation of cTnI and NT-proBNP were detected nor echocardiographic structural and functional abnormalities were found when compared with healthy control and risk factor-matched control, including those with myocardial injury in the acute phase. Our finding was consistent with previously published longer period follow-up studies. After a median interval of 6 months, echocardiographic measurements in COVID-19 survivors were not different between patients with and without myocardial injury during the acute COVID-19 phase (24). Combining our findings and previous follow-up results, it is suggested that myocardial injury and echocardiographic structural and functional abnormalities observed in the acute phase of COVID-19 infection might be reversible. The resolution of CMR abnormalities in COVID-19 athletes seems to be an example of this reversibility. In a consecutive follow-up study on athletes, CMR imaging revealed elevated T1, elevated T2, and late gadolinium enhancement in 2.3% of patients after a short interval (10–77 days) from diagnosis. However, a repeated CMR 4–14 weeks later from the first follow-up demonstrated resolution of T2 elevation in 100% and late gadolinium enhancement in 41% of patients (13). Thus, the cardiac abnormalities observed in COVID-19 survivors in previous studies (9, 12, 13) might be due to the short follow-up period and they might resolve in the long run. Another possible explanation for those observed persistent cardiac abnormalities in survivors could be the effect of pre-existing conditions in COVID-19 patients, such as hypertension, coronary artery disease, diabetes which are usually seen in the seniors. These patients tend to suffer more severe pneumonia (3), which further heavies the burden of the heart with mechanical ventilation. To avoid such confounders, COVID-19 survivors in our study were compared with a group of risk-factor matched control, with no significant cardiac abnormalities being found in the COVID-19 survivor group. Taken together, COVID-19 *per se* does not appear to cause long-term cardiac sequelae after recovery from acute illness.

The proposed mechanism of myocardial injury and dysfunction in patients with COVID-19 infection include cytokine-mediated damage, oxygen supply-demand imbalance, ischemic injury from microvascular thrombi formation, a direct viral infection of the myocardium, and pulmonary hypertension-induced RV dysfunction (4, 25, 26). The oxygen saturation was quite normal in COVID-19 survivors on the day of echocardiographic examination, lowering the possibility of oxygen supply-demand imbalance. Pulmonary artery systolic pressure in the COVID-19 survivors was also not different from that in healthy control. Previous studies have demonstrated that the cardiac structural and functional abnormalities caused by ischemic injury resolved after successful revascularization (27, 28). Longitudinal studies have demonstrated gradual declines of serum concentration of inflammatory biomarkers including IL-6, IL-8, tumor necrosis factor-α, and high-sensitivity C-reactive protein (hs-CRP) at the late stage of illness in COVID-19 survivors (29). Another study reported slight increased CRP levels in 16% of COVID-19 patients 2 months after symptom onset (30). Fulminant myocarditis is an inflammatory disease of the myocardium most often caused by a viral infection with severe impairment of LV systolic function in the acute phase. Previous reports showed that LV ejection fraction recovered at follow-up in survivors with fulminant myocarditis (31, 32). It is speculated that when the underlying pathogenic conditions were eliminated, the myocardial dysfunction would be reversed. Those findings in inflammatory biomarkers, oxygen saturation, and pulmonary artery systolic pressure in our study and previous studies (27, 28, 31, 32) support the observations in the present study that no significant differences exist in cTnI concentration, and echocardiographic structural and functional parameters among COVID-19 survivors, healthy control, and risk factor-matched control 327 days after diagnosis of COVID-19 infection.

Some limitations existed in our study. First, the quantitative echocardiographic data were unavailable at the onset of COVID-19 in isolation wards, which makes the longitudinal comparison of echocardiographic parameters impossible. Second, we did not perform segmental strain comparisons among groups. A previous study (33) has shown basal longitudinal strain dysfunction in COVID-19 patients in the acute phase of illness. Nevertheless, this study also showed decreased global longitudinal strain. Global longitudinal strain was calculated by averaging peak strains in 17 segments in our study. If one or several segment(s) has or have significantly decreased strain, the global longitudinal strain would be decreased concomitantly. Since no significant differences in global longitudinal strain were found in our study, we did not do further analysis in the segmental strain. Third, our study was based on a small sampling of survivors, thus, multicenter study with a larger population and longer follow-up period would be needed to provide more valuable information on the long-term cardiac consequences of COVID-19 infection.

## Conclusions

This study showed that COVID-19 survivors, including those with significantly elevated cTnI at admission and those with the severe and critical types of illness, did not have evident echocardiographic proof of cardiac structural and functional abnormalities 327 days after diagnosis.

## Data Availability Statement

The original contributions presented in the study are included in the article/supplementary material, further inquiries can be directed to the corresponding author.

## Ethics Statement

The studies involving human participants were reviewed and approved by the Tongji Hospital Ethics Committee. The patients/participants provided their written informed consent to participate in this study.

## Author Contributions

Y-BD, Y-NL, X-JB, H-YL, and YZ conceived and designed the study. Y-PG, WZ, P-NH, X-QC, R-YS, Y-NL, and Y-BD collected clinical and ultrasound data. Y-PG, WZ, LL, Q-YT, JZ, and JS analyzed data and performed the statistical analysis. Y-BD, Y-NL, Y-PG, and WZ drafted the manuscript. All authors approved the manuscript.

## Conflict of Interest

The authors declare that the research was conducted in the absence of any commercial or financial relationships that could be construed as a potential conflict of interest.

## Publisher's Note

All claims expressed in this article are solely those of the authors and do not necessarily represent those of their affiliated organizations, or those of the publisher, the editors and the reviewers. Any product that may be evaluated in this article, or claim that may be made by its manufacturer, is not guaranteed or endorsed by the publisher.

## Footnotes

^1^National Health Commission of the People's Republic of China. Diagnosis and treatment protocol of novel coronavirus (trial version 7th). National Health Commission of the People's Republic of China Website. http://www.nhc.gov.cn/yzygj/s7653p/202003/46c9294a7dfe4cef80dc7f5912eb1989.shtml. Accessed March 4, 2020.
